# Removal of black tattoos by Picosecond Q-switched Nd-YAG laser in the middle eastern skin type IV: prospective study

**DOI:** 10.1007/s10103-024-04140-w

**Published:** 2024-08-13

**Authors:** Mayada A. Ismail, Lamiaa H. Elgarhy, Ghada F. R. Hassan, Soha Abdallah Hawwam

**Affiliations:** Dermatology and Venereology, Faculty of Medicine, Tanta University, Tanta, El-Gharbia, Egypt

**Keywords:** Tattoos, Pico-laser, Skin of color, Middle eastern skin type IV

## Abstract

Tattoo removal is considered a challenging field in cosmetic dermatology. Picosecond Q-switched Nd-YAG lasers targeting unique chromophores effectively manage this condition without serious complications. To evaluate the efficacy and safety of Picosecond Q-switched Nd-YAG laser in the treatment of black tattoos in the skin of middle eastern mostly skin type IV. The study was carried out on 20 patients with skin type IV the most common in middle eastern area with professional black tattoos. They were treated by Picosecond Nd-YAG laser (2 sessions 8 weeks apart). The percentage of improvement ranged from 20.0 to 95.0 (with a mean of 61 ± 24.6). 8 patients (40%) showed excellent improvement, 4 patients (20%) showed marked improvement, 4 patients (20%) showed moderate improvement, and 4 patients (20%) showed mild improvement. No severe side effects were detected. Picosecond Nd-YAG laser was an effective and safe technique in the treatment of professional black tattoos; with only 2 sessions most patients reached excellent to moderate response with minimal side effects.

## Introduction

Tattoos have long been used to enhance beauty, signify belonging or punishment, and express an artistic perspective on the body, mostly common in Western countries [1]. The most common color used for tattoos is black; however, colorful tattoos that use multiple dyes have become increasingly popular. The demand for an effective method for tattoo removal, such as lasers, has also increased. Tattoo removal has become complicated because of the use of multiple colors in modern tattoos which is more difficult to remove [2].

Quality-switched (QS) lasers, which are capable of delivering short pulses of laser energy in the nanosecond domain, have been considered for decades the best devices for the selective removal of tattoos from the skin. Although the exact mechanism of laser tattoo removal is not completely understood, selective photothermolysis (SPTL) theory [3] is considered to be the primary principle of tattoo removal by lasers. Delivering a sufficient amount of energy to the target area (pigments in the tattoo) can destroy it with little or no damage to the surrounding tissues. The pulse durations should be equal to or less than the thermal relaxation time (TRT) of the target tissue. Therefore, pulse durations that are shorter than the target area’s TRT are important in cosmetic laser treatments of pigmentation therapy. However, the range of nanosecond-domain pulse duration is still too long to completely break the tattoo ink into smaller particles. Most tattoo pigments have a TRT in the range of picoseconds since the size of tattoo particles ranges between 40 and 300 nm in vivo [4, 5].

### Objective

Evaluation of the efficacy and safety of Picosecond Nd-YAG laser in the treatment of professional black tattoos in the skin of middle eastern patients mostly skin type IV.

## Patients and methods

### Patients

This study was a prospective simple randomized study carried out on 20 middle eastern patients with skin type IV with professional black tattoos, after approval of the research ethics committee of the Faculty of Medicine, Tanta University (approval code 32,990/11/20). This study was approved by the Research Ethical Committee following the Declaration of Helsinki (2008 version). This trial followed the CONSORT guiding principles. Patients included in this study didn’t receive any previous treatment for their tattoos before this study and are accepted to be enrolled in the study. Informed consent was signed by all patients (informed consent to discuss their data and informed consent to publish their pictures). Patients with renal insufficiency, liver disease, history of keloidal scarring, photosensitivity, or using photosensitive drugs as well as pregnant and lactating females were excluded from the study. All patients were subjected to complete history taking, and thorough general and dermatological examination.

Digital photographs were taken before the procedure and after every session of laser by using (Sony Cyber-shot DSCW690 16.1 MP 10× optical zoom digital camera; Sony, Japan).

## Methods

### Laser procedure

All patients applied a topical anesthetic cream; prilocaine 2.5% plus lidocaine 2.5% (Prila 5%, Avalon Pharma) 30 min before the session on the affected area. This topical cream was washed off with soap and water thoroughly before the procedure. Protective goggles were properly placed on the patient’s eyes.

**Fig. 1:**
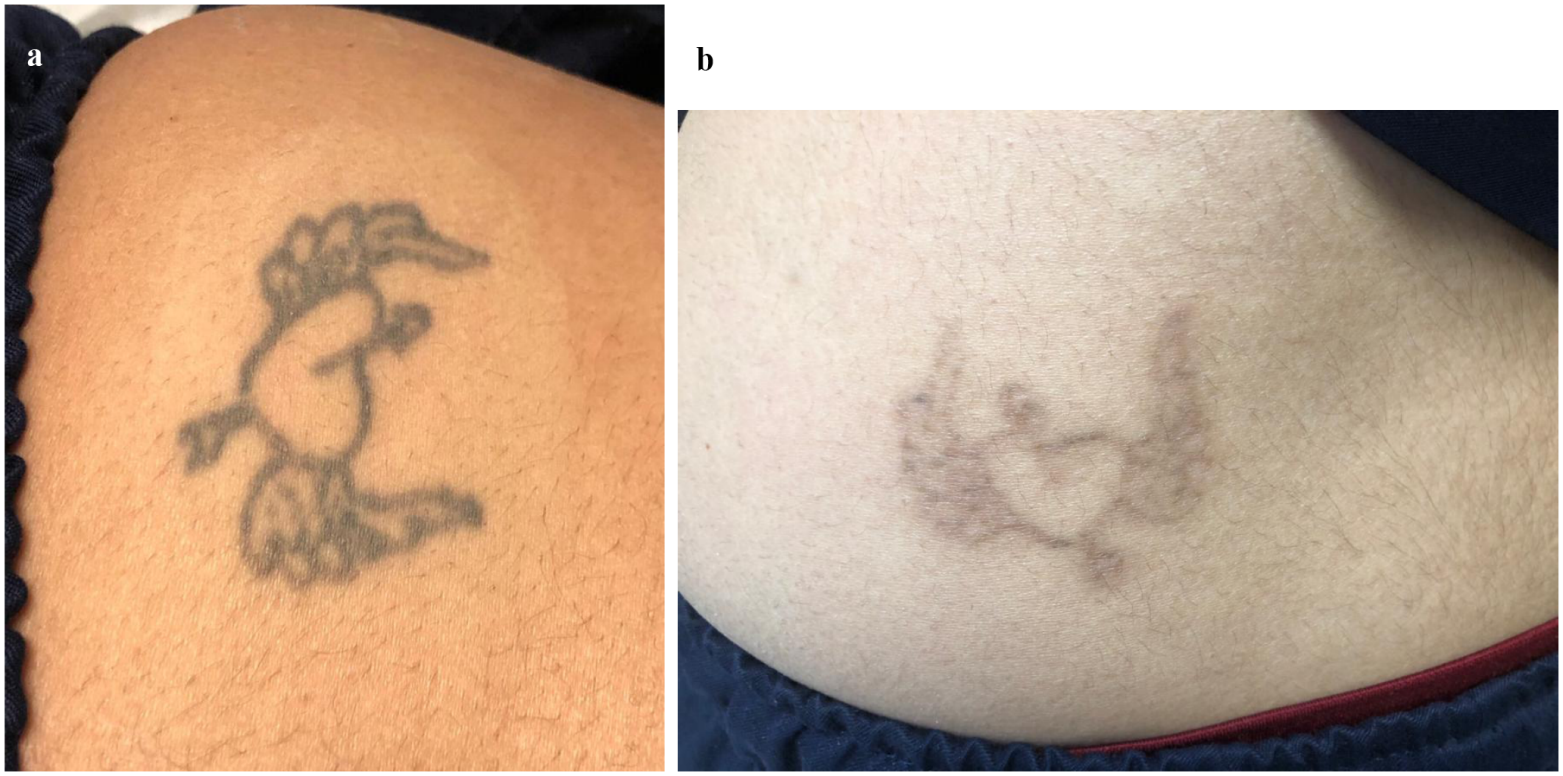
**(a)** A 45-year-old female patient with a black tattoo on the thigh. **(b)**: Showed excellent improvement of the black tattoo, after two sessions of picosecond 1064 nm Nd: YAG laser 8 weeks apart

**Fig. 2:**
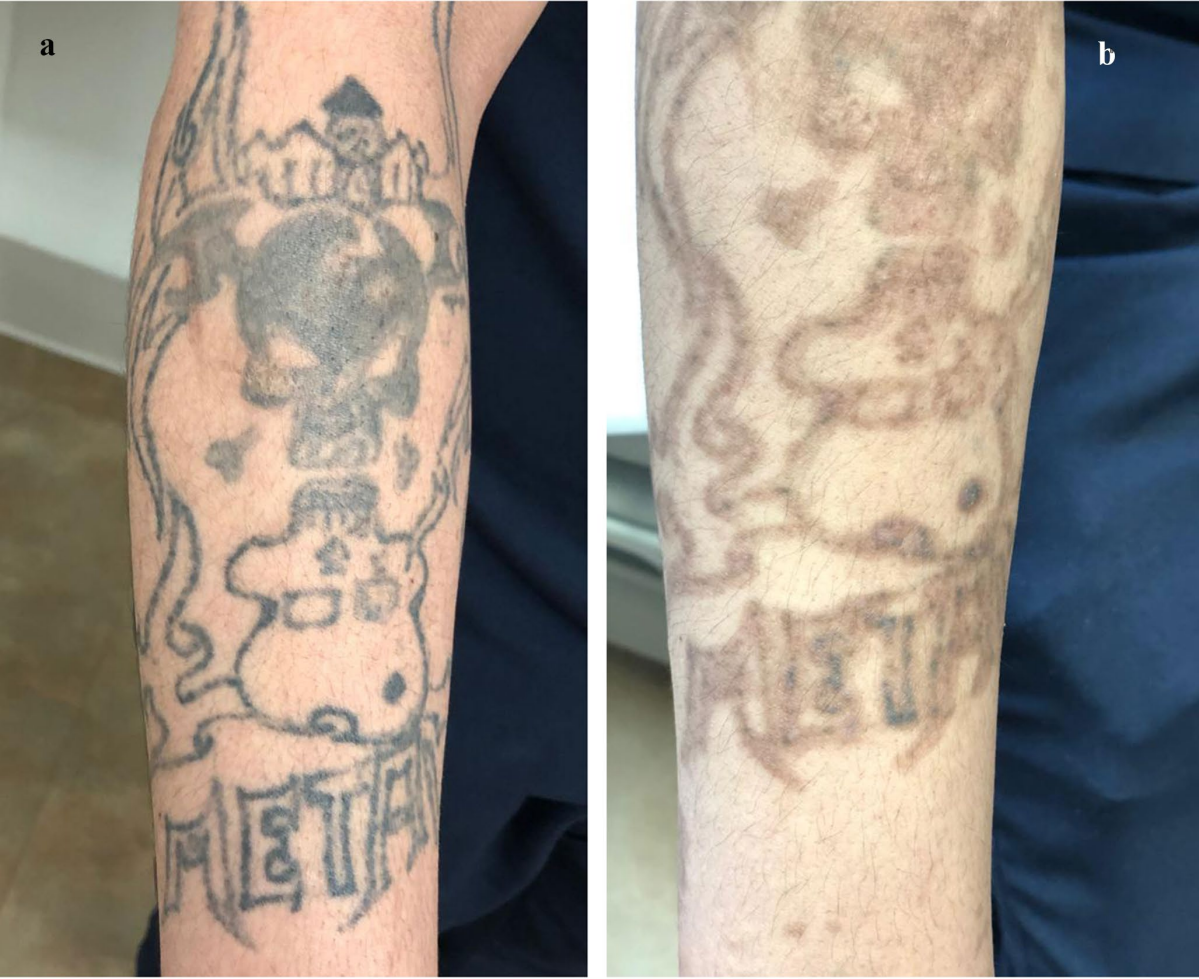
**(a)**: A 30-years male patient with a black tattoo on the right arm. **(b)**: Showed marked improvement of the black tatto, after two sessions of picosecond 1064 nm Nd: YAG laser 8 weeks apart (8 weeks after the last session)

The laser procedure was performed with picosecond 1064 nm Nd: YAG laser (PicoWay, Syneron, Candella, USA for the treatment of professional black tattoos. Each laser session was performed with a single pass without overlap with the zoom handpiece with a spot size of 2, 3 mm, fluence of 4 to 5 J/cm2, pulse duration of 450 picoseconds (ps), and repetition rate of 1–2 Hz. The parameters were adjusted based on the size of the tattoo and the patient’s skin type as shown in (Figs. 1a and 2a). Every patient received two sessions, 8 weeks apart. Post-procedure cold compresses were used to minimize discomfort for about 30 min; patients were advised to use topical antibiotic cream on the target area after each laser treatment session.

### Post-treatment care

The patients were educated to avoid direct sunlight and recommended to apply sunscreen agents between the laser treatment sessions.

### Assessment of the efficacy of the therapeutic procedure

1. Clinical evaluation

- Global Aesthetic Improvement Scale (GAIS) [6]: Three blinded dermatologists assessed the photos of the patients before treatment and 4 weeks after the last session as follows: No improvement, < 25% improvement (mild improvement), 26–50% (moderate improvement), 51–75% (marked improvement), and > 75% (excellent improvement).
- Patient’s satisfaction: Each patient was asked at the final visit (4 weeks after the last session) about his /her satisfaction according to whether the patient was unsatisfied, slightly satisfied, satisfied, or very satisfied.
- All patients were informed to report any complaints or complications that occurred as erythema, pain, infection, hyperpigmentation, and edema.

### Statistical analysis

Data were fed to the computer and analyzed using IBM SPSS software package version 20.0 (v 16; SPSS Inc., Chicago, IL, USA).

## Results

The clinical data of the patients is demonstrated in Table (1).

1. Clinical evaluation

- **Evaluation of efficacy of treatment according to the percentage of improvement and GAIS**: The percentage of improvement ranged from 20.0 to 95.0 with a mean of 61 ± 24.6 (median 65%). 8 patients (40%) showed excellent response as shown in (Figs. 1b and 2b), 4 patients (20%) showed marked response, 4 patients (20%) showed moderate response, and 4 patients (20%) showed mild response (Table 1).
- **Evaluation according to the patient`s satisfaction**: 10 patients (50%) were very satisfied, 5 patients (25%) were satisfied, and 5 patients (25%) were slightly satisfied, with no unsatisfied patients (Table 1).
- There were no significant relations between the percentage of improvement and sex or site of tattoos, also there was no significant correlation between the percentage of improvement and either age or duration of tattoo formation (Tables 2 and 3).
- **Evaluation of side effects of the treatment in both groups**: Pain during sessions occurred in all patients (100%) but it was mild to moderate. Erythema for one day occurred in all patients (100%) but only 4 patients complained of persistent erythema for three days (20%). Petechiae were noticed post-laser in all patients (100%). Edema was found in 16 patients (80%), and it disappeared within one day.

**Table 1:** Distribution of the studied patients according to different parameters (*n* = 20)

	No. (%)
Sex	
Male	8 (40%)
Female	12 (60%)
**Age (years)**	
Mean ± SD.	35.1 ± 9.9
Median (Min. – Max.)	31.5 (22–55)
**Skin type**	
II	2 (10%)
III	4 (20%)
IV	14 (70%)
**Site**	
LL	2 (10%)
Back	8 (40%)
Forearm	4 (20%)
Hands	2 (10%)
Face	4 (20%)
**Degree of improvement**	
Mild	4 (20%)
Moderate	4 (20%)
Marked	4 (20%)
Excellent	8 (40%)
**Percent of improvement**	
Mean ± SD.	61 ± 24.6
Median (Min. – Max.)	65 (20–95)
**Duration (years)**	
Mean ± SD.	6.7 ± 2.2
Median (Min. – Max.)	6.5 (4–10)
**Number of sessions**	
2	20 (100%)

**Table 2:** Correlation between percentage of improvement with age and duration (*n* = 20)

	*R*	*P*
Percent of improvement vs.		
** Age (years)**	0.117	0.624
** Duration (years)**	0.356	0.124

r: Pearson coefficient

**Table 3:** Relation between percentage of improvement with sex and site (*n* = 20)

	No.	Percent of improvement		Test of Sig.	*P*
		Mean ± SD.	Median (Min. – Max.)		
Sex					
Male	8	58.8 ± 17.9	55 (40–85)	t = 0.326	0.748
Female	12	62.5 ± 28.9	75 (20–95)		
**Site**					
LL	2	30 ± 0	30 (30–30)	F = 1.524	0.245
Back	8	61.3 ± 30.8	65 (20–95)		
Forearm	4	72.5 ± 14.4	72.5 (60–85)		
Hands	2	80 ± 0	80 (80–80)		
Face	4	55 ± 17.3	55 (40–70)		

SD: Standard deviation t: Student t-test F: F for One way ANOVA test

p: p value for comparing between the studied categories

## Discussion

Picosecond lasers, with a pulse duration of 10^–12^ seconds, are considered a fast and effective method for tattoo removal because most tattoo pigment size ranges from 30 to 300 nm with short thermal relaxation time (< 10 ns) [7]. Therefore, a picosecond laser can provide greater thermal stress in targeted tattoos [8]. In addition to photoacoustic effects within targeted tattoos leading to the mechanical dissolution of the ink particles, an endothermic steam carbon reaction occurs, which changes the properties of inks, reducing their visibility [9, 10]. Furthermore, picosecond technology could permit lower fluences to be delivered, which theoretically lower the risk of adverse effect [11].

In a study by Park et al. 2021, they included only nine patients for tattoo removal in their study (using picosecond Nd: YAG laser). They reported that the mean clearance was 86.6% after receiving 6–8 sessions with 4–8 weeks in-between, they used fluence 2.5–4.8 j/cm^2^, repetition rate 6–10 Hz). This was in accordance with the current study with more patients (20 patients) and better results (we reported that 40% of patients showed excellent improvement, the mean of clearance was 61 ± 24.6) with only two sessions 8 weeks apart [12].

Ross et al. were the first to report the better effect of the picosecond laser comparing nanosecond laser in human patients using a 1,064-nm Nd: YAG laser with a pulse duration of either 35 picoseconds or 10 nanoseconds. The parameters included a fluence of 0.65 J/cm2 and a spot size of 1.4 mm [4]. Choi et al. compared picosecond and nanosecond lasers in their ability to remove multi-colored tattoos using Hartley guinea pig. They first compared a nanosecond quality-switched Nd: YAG laser with picosecond alexandrite and quality-switched Nd: YAG lasers and then the picosecond quality-switched Nd: YAG laser with the picosecond alexandrite laser and concluded that picosecond lasers are more effective and safer than nanosecond laser [14].

Adverse effects of pico-laser included pain, swelling, and blistering, but resolved within a few days. Also, hyperpigmentation or hypopigmentation can occur, as reported as 20% of hypopigmentation and 13% of hyperpigmentation in a previous study using picosecond alexandrite laser [15]. In the present study, pain, erythema, petechiae, and swelling were the only reported side effects which resolved within one to three days.

The main mechanism of picosecond laser tattoo removal involves the fragmentation of the chromophore through both photothermal and photoacoustic effects. Picosecond lasers transmit light pulse lengths that are closer to the TRT of tattoo pigment molecules; therefore, they can deliver heat radiation more efficiently and can be destructively focused on the target area. For example, the average size of carbon black in Indian ink is approximately 40 nm in diameter, while the TRT for 40 nm particles is approximately one nanosecond. The picosecond pulse can be thermally confined to the target because it is irradiated with a pulse duration of less than 1 nanosecond [14].

Bennardo et al. conducted their study on 34 patients, The patients received seven sessions (mean 3.3 + 2.0 sessions), more sessions were done for the professional admixed colored tattoos in extremities while facial tattoos and one colored black tattoo responded to fewer sessions, the sessions were done with 8 weeks interval, they reported over 40% of patients showing complete removal (80–100%) of tattoos and the rest were with 60–80% removal. No severe side effects were reported. No patients left the study. In three cases, a final “ghost effect” was reported; six participants developed petechiae after treatment and were medicated with an occlusive dressing, hydrolytic enzymes, and antibiotics, with the disappearance of the vascular manifestations in one to two weeks [16]. This was in agreement with the current study but without the ghost effect.

Picosecond pulses seem to guarantee higher effectiveness than longer nanosecond pulses, thus reducing the number of treatment sessions and improving cosmetic outcomes. The picosecond Nd: YAG laser is reported as the optimal tattoo removal device because it can obtain excellent cosmetic results with minimal skin damage, low scarring risk, and lower incidence of hypopigmentation than the picosecond alexandrite which causes more side effects. A two-month interval between sessions permits the removal of tattoo-pigmented particles via the phagocytic cells, and it is necessary before carrying out any other session or procedure. The results, however, may not be always cosmetically acceptable [18, 19]. However, these results may be biased because picosecond sources are often used for harder-to-treat tattoos, such as professional tattoos with mixed colors [16].

## Conclusions

The exact mechanisms of laser-assisted tattoo fragmentation are hardly known. Different factors, such as short and intense laser pulses, non-linear effects of light, and nonlinear thermal properties in tattoo particles may play a crucial role. Q-switched laser treatments may be considered the gold-standard therapy. Theoretical considerations assumed that shorter pulse durations allow for more effective fragmentation of tattoo particles, and initial studies affirm the effectiveness of picosecond pulses in tattoo treatment, with a reduction in pain and side effects. Picosecond lasers demonstrate a greater ability to degrade smaller tattoo pigments through the photoacoustic effect. Picosecond lasers are a promising technology with the potential to optimize the treatment of tattoos.

Picosecond Nd-YAG laser was an effective and safe technique in the treatment of black tattoos in the middle eastern skin type mostly skin type IV patients with only 2 sessions most patients reached excellent to moderate response. Picosecond lasers may achieve a satisfactory result with fewer sessions and side effects.

The limitations of this study are the small number of participants, and the lack of treating darker skin types as V, VI and the lack of comparing picosecond and nanosecond laser sources and comparing picosecond Nd: YAG and picosecond alexandrite lasers which are recommended in further studies to determine the best treatment for tattoos in dark skin types.
